# Tolerance in Organ Transplantation: From Conventional Immunosuppression to Extracellular Vesicles

**DOI:** 10.3389/fimmu.2014.00416

**Published:** 2014-09-17

**Authors:** Marta Monguió-Tortajada, Ricardo Lauzurica-Valdemoros, Francesc E. Borràs

**Affiliations:** ^1^Innovation in Vesicles and Cells for Application Therapy Group (IVECAT), Institut d’Investigació Germans Trias i Pujol, Badalona, Spain; ^2^Nephrology Service, Hospital Universitari Germans Trias i Pujol, Badalona, Spain

**Keywords:** exosomes, extracellular vesicles, transplantation immunology, tolerance, graft rejection, graft survival, organ transplantation

## Abstract

Organ transplantation is often the unique solution for organ failure. However, rejection is still an unsolved problem. Although acute rejection is well controlled, the chronic use of immunosuppressive drugs for allograft acceptance causes numerous side effects in the recipient and do not prevent chronic allograft dysfunction. Different alternative therapies have been proposed to replace the classical treatment for allograft rejection. The alternative therapies are mainly based in pre-infusions of different types of regulatory cells, including DCs, MSCs, and Tregs. Nevertheless, these approaches lack full efficiency and have many problems related to availability and applicability. In this context, the use of extracellular vesicles, and in particular exosomes, may represent a cell-free alternative approach in inducing transplant tolerance and survival. Preliminary approaches *in vitro* and *in vivo* have demonstrated the efficient alloantigen presentation and immunomodulation abilities of exosomes, leading to alloantigen-specific tolerance and allograft acceptance in rodent models. Donor exosomes have been used alone, processed by recipient antigen-presenting cells, or administered together with suboptimal doses of immunosuppressive drugs, achieving specific allograft tolerance and infinite transplant survival. In this review, we gathered the latest exosome-based strategies for graft acceptance and discuss the tolerance mechanisms involved in organ tolerance mediated by the administration of exosomes. We will also deal with the feasibility and difficulties that arise from the application of this strategy into the clinic.

## Introduction

Solid organ transplantation (SOT) is the unique solution for end-stage organ failure, and can be considered among the major accomplishments of the twentieth century in human health. Only in 2012, it is estimated that about 115,000 solid organ transplants were performed worldwide (1). Apart from saving lives, SOT is a cost-effective alternative to other medical options (when available). For instance, it is well established that kidney transplantation (by far the most transplanted organ worldwide, being 65% of total SOT), increases survival rates, guarantees a better quality-of-life and it is also less costly in the long term compared to hemodialysis.

The improved methodology in surgical techniques, technological advances, and research in biological and pharmaceutical products have profoundly improved the survival of transplanted patients. Remarkably, the maximum survival reported for a kidney transplanted patient is 46 years, and 39 years for a liver transplanted patient (2). However, the overall data indicates that the median graft survival of kidney transplants is 50% after 10 years. Most of these graft looses are due to chronic rejection episodes conducted by the recipient’s immune system against the graft. Therefore, one of the most important challenges in organ transplantation is achieving graft immunological tolerance, i.e., preventing the recipient’s immune system attack and destruction of the transplanted organ leading to graft rejection.

To prevent graft rejection, immunosuppresive drugs (ISd) have been successfully given to transplant recipients. Not in vain, these ISd are responsible for the increased survival of transplanted patients. Nonetheless, the chronic use of ISd leads to drug related-toxicity and to an un-specific and general suppression of the immune system, which may cause rising of opportunistic infections and malignancy. Therefore, alternative treatments to classical immunosuppression to induce donor-specific tolerance need to be found. In this review, we will briefly mention the mechanisms of graft rejection, the classical immunosuppression strategies, and how new extracellular vesicles (EVs)-based strategies may be an opportunity to induce organ tolerance. We will also discuss some critical points to be solved for the application of this strategy into the clinic.

## Graft Rejection

Transplant rejection is a complex immune response directed against the alloantigens (antigenic alleles) specifically expressed by the graft, which are recognized as “non-self” by the host’s immune system. These alloantigens essentially include the major histocompatibility complex (MHC) molecules and also minor histocompatibility antigens (miHAs) expressed by graft cells. The final outcome of this immune response is the rejection of the organ, leading the recipient to a new transplantation or alternative replacement therapy, such as dialysis in the case of kidney failure.

According to the clinical and pathological course, graft rejection may be classified as (3) (i) Hyperacute rejection, which takes place after only few minutes to few hours of transplantation; (ii) Acute cellular rejection, mainly mediated by cells and occurring within a few weeks; (iii) Acute humoral rejection, arising at the first/second week after transplantation and mediated by antibodies directed against alloantigens; (iv) Chronic rejection, which may appear at any time (even years) after the acute phase and progressively deteriorates the graft function. This last type of rejection is responsible of most of rejected organs after 1 year from transplant.

In all graft rejection types, the effector mechanisms responsible for injury and destruction of the transplanted organ involve the participation of all (cellular and soluble) components of the immune system. These include not only T and B lymphocytes, the major effectors of the adaptive immune system (involved in antigen-specific rejection), but also cells of the innate immune system including endothelial cells, NK cells, macrophages, and/or polymorphonuclear cells. Also, antigen-specific (antibodies) and un-specific (complement) soluble molecules of the immune system are involved in the host’s attack to the graft cells. A recent review provides an excellent overview of the cellular and molecular mechanisms leading to graft rejection (4).

## Alloantigens, the Inducers of Graft Rejection

As mentioned above, MHC molecules from donor origin are the main molecular targets triggering the immune attack suffered by the graft. MHC molecules are a high variable, codominantly expressed, and autosomically inherited genes expressed by most cell types (5). Physiologically, MHC molecules (HLA molecules in humans) are the essential bridge between innate immunity and adaptive (specific) antigenic responses. In short, antigen-presenting cells [APCs, such as dendritic cells (DCs) or Macrophages] capture pathogens and exhibit pathogen-derived processed antigens via MHC molecules to antigen-specific T cells. This recognition initiates both cellular and humoral adaptive immune responses, ideally leading to the eradication of the infective process.

**Figure 1:**
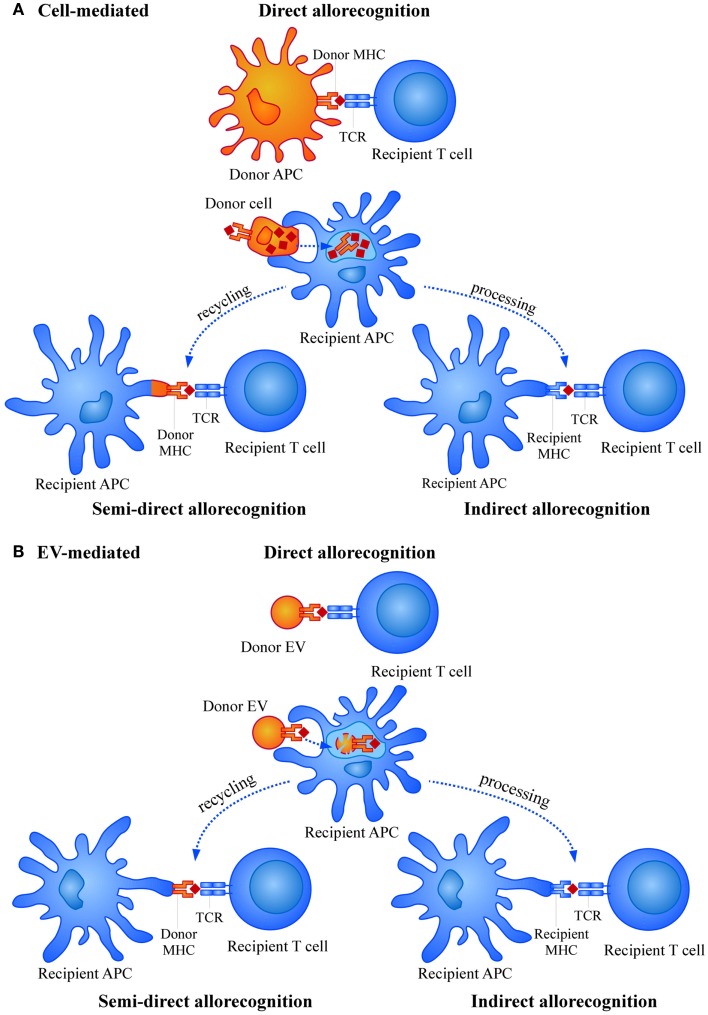
**Mechanisms of allorecognition by (A) cells and (B) extracellular vesicles (EVs)**.

In the transplant situation, graft cells expressing donor HLA molecules are recognized by the recipient T cells as “non-self” molecules, leading to a similar induction of the immune response (6). However, while in a “physiological” immune response against a pathogen, the number of activated T cell clones is rather low (approximately 1/100,000), in the transplant situation this number is increased to 1/100 or even more. Thus, the potency of inducing immune responses by HLA foreign alloantigens is much higher compared to a conventional immune response. This strong allorecognition is mainly based in two different aspects. First, the high level of polymorphism associated to HLA genes, the most polymorphic loci described in humans, that is continuously updated with the appearance of new allelic forms (7). Second, the wide repertoire of T cells able to respond to the allostimulation. This is due to the fact that priming and activation of T cells by alloantigens may occur through three different mechanisms, namely direct allorecognition, semi-direct allorecognition, and indirect allorecognition (Figure 1A) (8–11). In the direct allorecognition, recipient T cells “directly” recognize donor peptide-MHC complexes on donor APCs. This mechanism is responsible for the acute rejection and diminishes with time due to the progressive loss of donor APCs. Importantly, in acute rejection the inflammation caused in the organ by the surgical procedure or the period of ischemia-reperfusion may induce the expression of MHC and adhesion molecules (for instance in endothelial cells) and also the production of other inflammatory mediators that contribute to amplify the immune attack. The semi-direct allorecognition occurs when donor MHC molecules are recycled and presented as intact molecules on recipient APCs and presented to antigen-specific T cells (12). Finally, in the indirect allorecognition, recipient APCs capture and process donor alloantigens (as any exogenous antigen in a “physiological” immune response) and the derived peptides are exposed to T cell recognition via self (of the recipient) MHC molecules. Both, semi-direct and indirect allorecognition may be involved in chronic rejection.

Of course, an ideal situation to avoid the immune response against alloantigens would be to find a complete HLA compatible donor, a possibility reduced to HLA-identical siblings. Considering the multiple combinations and possibilities derived from the number and diversity of MHC alleles and miHAs, it is extremely difficult to find a high level of HLA compatibility between a donor and a recipient. Thus, SOT has been routinely performed between ABO compatible donor–recipient pairs with no evidence of preformed anti-HLA antibodies (Cross-match negative). However, due to the shortage of organs from deceased donors, an important number of SOTs (mainly kidney transplantations) are performed over HLA or ABO antibody barriers. Desensitization strategies such as plasmapheresis, immune adsorption, anti-CD20 antibodies, and the use of complement inhibitors, may help those end-stage kidney disease patients (13). Yet, recipients with high titters of antibodies and HLA sensitized patients demonstrate a limiting long-term run.

The necessity to effectively block the immune response against the graft and therefore avoid transplant rejection has encouraged the study and generation of different immunosuppressive strategies.

## Current Approaches to Immunosuppression

Clinically, immunosuppression (IS) in transplantation has the mission to prevent and treat acute rejection and to avoid chronic graft injury. These main objectives have to be in fine equilibrium with minimal adverse effects for the patient. Needless to say this balance is very difficult to achieve.

During the early years of organ transplantation, basic IS used corticosteroids and azathioprine. The appearance of Cyclosporine introduced a significant change in the field of transplantation, since the use of this drug dramatically reduced acute rejection episodes. Cyclosporine became the basic ISd until a new calcineurin inhibitor, Tacrolimus, was introduced in the 90s. Tacrolimus was more powerful compared to Cyclosporine, but shared a similar spectrum of adverse effects (basically worsening cardio-vascular profiles). Tacrolimus is still today the drug of reference for renal transplantation. When used in combination with antiproliferative drugs such as mycophenolate mophetil/mycophenolate sodium, the frequency of acute rejection episodes was set below 20%. The “Symphony” international study established the guidelines for IS in transplantation for the last 15 years. Following the study, more than 85% of current IS protocols are based on a combination of steroids, tacrolimus at low doses plus mycophenolate (14).

A new generation of ISd emerged with the synthesis of mTOR inhibitors, sirolimus, and everolimus. These drugs were initially applied in the so-called “no nephrotoxicity” protocols, which avoided using calcineurin inhibitors. However, a high frequency of adverse effects combined with increased rates of acute rejection episodes forced to stop treatments. Today mTOR inhibitors are combined with reduced doses of calcineurin inhibitors (15). Several independent studies support this strategy, although a large cohort study showing their efficacy and acceptable side effects is still missing.

Biological-derived drugs are the next generation of “conventional” IS. One of the most promising new drugs is Belatacept, a human fusion protein aimed to block the co-stimulation of T lymphocytes. Several studies suggest an efficacy comparable to calcineurin inhibitors, but preserving organ function (lower incidence of chronic kidney disease). Although Belatacept shows a good cardio-vascular profile (being this the main complication of Tacrolimus), an undesirable side effect is an increased incidence of post-transplant lymphomas, especially in Epstein-Barr virus seronegative patients. This treatment is therefore not used in this subgroup of patients (16, 17).

Many other agents are being evaluated in clinical trials to prevent acute rejection. Some examples are Sotrastaurine (potent and selective inhibitor isoform of protein kinase C), Tofacitinib (selective inhibitor of JAK 3 kinase), Alefacept (anti-CD2 humanized monoclonal antibody), and others (18–20). However, most of these studies are discontinued due to lower efficacy and safety profiles when compared to conventional ISd.

Most of the improvements and new drugs show efficacy in the short term after transplantation, thus controlling acute rejection (the first objective). Unfortunately, similar results have not been observed at long-term (21). The so-called chronic graft dysfunction is still a major cause of graft loss in kidney transplant [chronic kidney dysfunction (CKD)]. It is estimated that a 4% of transplanted kidneys are lost every year by this pathological process. In fact, CKD may be considered an epidemic itself, with similar prevalence to diabetes in the general population (22). Thus, the second mission of conventional IS aiming to control chronic graft injury is still not fully accomplished.

Another unsolved issue with ISd is the reduction of adverse side effects largely associated with these drugs. Remarkably, the main cause of loss of fully functional transplanted kidneys is due to death of the recipient patients affected by ISd side effects, including cardio-vascular diseases, opportunistic infections, and post-transplant neoplasia (23–25).

In summary, the use of ISd has markedly reduced the incidence of acute rejection and early graft loss. However, the numerous adverse side effects observed, and failure to effectively prevent chronic allograft dysfunction of conventional IS boosted the development of alternative strategies to avoid graft rejection.

## New Approaches for Immune Regulation: Cell Therapy

Among the new approaches for the induction of allograft tolerance, the use of the regulatory properties of different cell types, such as regulatory T cells, DCs, and mesenchymal stem cells (MSCs) has been evaluated in animal models and also in some clinical trials using primates, with promising results (26, 27).

Regulatory T (Treg) cells have been widely studied for their capacities to modulate the immune response toward tolerance in different immunological contexts, being autoimmune diabetes the first to be considered for regulatory T cell therapy in a clinical trial (28). Their use in allograft acceptance has shown encouraging results (29–31), although no long term allograft tolerance has been attained so far. The mechanisms by which Treg cells manage to induce allograft tolerance are yet to be fully elucidated. Membrane-bound TGF-β and CTLA-4 expression is thought to mediate contact-dependent immunosuppression toward APCs and effector cells (32–35). Treg cells have been also reported to block the induction of IL-2 in T cells at the transcription level, leading to low proliferation and decreased activation of effector CD4^+^ and CD8^+^ T cells (36). Indirect recognition is thought to be the main allorecognition pathway suppressed by Treg cells as tolerance is deficient in animals lacking this mechanism of alloantigen presentation (37).

Different types of strategies have been tried to expand and activate Treg cells to enhance their immunosuppressive functions, thus several cytokine cocktails and immunosuppressants have been used to obtain more potent suppressor cells *ex vivo*, such as rapamycin (35). While some studies ensure IFN-γ induce Treg cells *in vitro*, other relate TGF-β and IL-10 as the most potent cytokines for Treg activation and survival (34, 38–42). On the other hand, there are reports showing the need for naïve T cells co-culture for obtaining alloantigen-specific Treg cells as the last depend on cytokines produced by ongoing Th1/Th2 immune response to develop (43). Anyway, the presence of CD4^+^ CD25^+^ T cells in the recipient is necessary to induce tolerance and achieve allograft acceptance (44).

Mesenchymal stem cells are an adult source of progenitor cells with the ability to self-replicate and differentiate to multiple lineages. MSCs have been proposed for their application in therapy of multiple diseases involving aberrant immune responses given their intrinsic immunoregulatory capabilities (35, 45–47) and ability to stimulate tissue repair and regeneration, as detailed further in the article by de Jong et al. published in this same issue. Treatment of injuries and diseases produced by unwanted inflammatory processes has been done with MSCs, and they have proven effective in kidney and cardiac injuries and in clinical trials for the treatment of Chron disease, graft versus host disease (GvHD), and diabetes (48, 49).

Mesenchymal stem cells are found to have different immunological policing according to the inflammatory milieu they are found in. For instance, under non-inflammatory conditions, MSCs promote regeneration and tissue repair, and have poor intrinsic immunogenicity due to the low expression of MHC or activation accessory molecules, which makes them suited to be used allogeneically in therapy and administered repeatedly (49, 50). On the other hand, MSCs become highly immunosuppressive when triggered by the inflammatory cytokines IFN-γ and TNF-α, and then importantly express MHC molecules (45, 48, 51), a combination that would be optimal for the purpose of achieving allograft tolerance.

In the transplantation set-up, there are some opposing studies (52–54), but the use of MSCs has been found also to help prolong heart and skin allograft survival and proves effective against refractory GvHD (45, 48). Relevantly, Ge et al. demonstrated the need of the right concomitant immunosuppressive for MSC engraftment and thus consecution of infinite allograft tolerance and also showed Ag-specific tolerance induction regardless of MSCs origin (55). A beneficial effect of MSC on experimental chronic graft nephropathy has been also reported in a rat kidney allograft model (56). Importantly, MSCs have been already used in clinical trials for allograft acceptance in the context of kidney transplantation, showing interesting results (57).

However, MSCs therapy has obvious concerns, which cannot be ignored, specifically their intrinsic tumorigenic potential given their self-replicating and differentiation capabilities. Moreover, their autologous use requires surgical intervention in compromised patients and exogenous expansion cultures, in which MSCs could modify their potency, efficiency, and safety (49).

Antigen-presenting cells have also been tested in the induction of tolerance. Among professional APCs, DCs actively participate in the physiological mechanisms of tolerance, through the induction of T cell anergy, depletion of antigen-specific T cells, and/or the promotion of regulatory T cells (Treg) (58–60). The tolerogenic potential of modified tolerogenic DCs (Tol-DCs) loaded with relevant antigens opened the possibility to treat certain autoimmune diseases in which antigenic proteins are at least partially characterized, such as multiple sclerosis or rheumatoid arthritis (61, 62). Similarly, the use of Tol-DCs has been tested in transplantation. Preliminary experiments in rodents have widely demonstrated that administration of different types of Tol-DCs prolong graft survival in combination with suboptimal doses of conventional immunosuppressant [reviewed in Ref. (63)]. As Tol-DCs may be obtained under GMP conditions (64), they are being used in clinical trials of tolerance induction in arthritis and allergy (www.clinicaltrials.gov) (65).

Some evidences support that unloaded Tol-DCs are sufficient for inducing allograft acceptance. In fact, it has been reported that recipient DCs are actually responsible for alloantigen presentation and tolerance attainment rather than injected DCs, from which they would capture alloantigens in a tolerogenic fashion (66). This would open the possibility to explore alternative cell-free alloantigen sources.

## Alloantigens: Opportunities in Tolerance Induction in Transplantation

Several sources of alloantigenic material have been investigated for the induction of tolerance in transplantation. Immunodominant peptides were first explored for the induction of tolerance (67), which demonstrated the specificity of the response. In the case of SOT, due to the high variability associated to MHC antigens, it is virtually impossible to synthesize and cover all the antigenic polymorphisms of a given donor–receptor pair, thus pointing to the need of finding specific sources of these alloantigens in each transplant situation. Mimicking the experiments performed in DC-based tumor immunotherapy (68), cell-free lysates were initially chosen as a source of alloantigens, showing some encouraging results (69–73).

Apoptotic bodies from donor origin have been also proposed as another alloantigen source given their immunoregulatory capabilities and enhanced capture by APCs [reviewed by Ref. (74)]. Moreover, apoptotic lymphocytes would be a rich MHC source (75, 76), easy to prepare, and also would not require pre-loading DCs *in vitro* as their use *per se* has been proven sufficient. They have been used in transplant models in mice and rats, showing a prolonged allograft survival, promoting donor-specific tolerance, and proving to be safe by intravenous administration (75–79). Significantly, these studies highlighted the importance of the right timing of the therapy and demonstrated apoptosis’ intrinsic immunoregulatory capabilities, as necrosis did not show the same beneficial effects (76). Nevertheless, despite the promising results shown by several groups, infinite survival of the allograft has not been attained yet. Also, given the content in damaged DNA and high heterogeneity, other sources of alloantigens, such as EVs, are being considered. In contrast to cell lysates and apoptotic bodies, EVs represent a more stable and controlled source, can be cryopreserved and produced for clinical purposes (80, 81).

## EVs as Alloantigens

Extracellular vesicles include a wide variety of lipid bilayered vesicles secreted by cells, ranging from nano to micrometric sizes and bearing distinct biochemical and physical properties. EVs mediate communication by transferring proteins and RNA between cells (82–84) not only at the paracrine level but also systemically. These vesicles are found in biological fluids like urine, blood, ascites fluid, cerebrospinal fluid, or semen [reviewed in Ref. (85)]. The term EVs refers to a broad spectrum of vesicles from different cell origin, biogenesis, function, and isolation method (86–88). Actually, most studies performed until now in this field refer to EVs as exosomes. While microvesicles are budded from the plasma membrane itself, exosomes are shed by many cell types upon the fusion of the multivesicular bodies (MVBs) with the plasma membrane and contain representative molecules from the cell they originate from, with functional proteins and RNA specifically sorted into them (89–91). Exosomes consistently express MHC antigens (92) and their composition is more homogeneous compared to apoptotic bodies (80) and less prone to inflammation compared to cell lysates. For this reason, exosomes, and extendedly EVs, have been proposed as a possible source for alloantigen presentation to the host.

Alloantigen presentation *in vivo* could be either directly mediated by the peptide-loaded MHC molecules found in the EVs or indirectly upon the capture and presentation by recipient APCs. Also, entire donor MHC molecules could be recycled by recipient’s APCs and presented to the recipient T cells (Figure 1B). There has been some controversy regarding the feasibility of direct presentation by EVs. Some studies proved the need of indirect presentation by DCs for exosomes to be able to stimulate T cells (93–95), while other groups did demonstrate direct functional presentation through exosomes themselves (96, 97).

## Source of EVs for Therapeutic Use

### Plasma EVs

Donor EVs containing MHC and miHAs may be obtained from multiple sources, each possessing intrinsic characteristics and advantages and being studied independently as strategies for allograft acceptance. The first biological fluid coming to mind given its ease of obtaining would be plasma. However, previous studies showed the little content of EVs and low MHC expression in healthy human plasma samples (98, 99), meaning plasma would not be the first choice in terms of alloantigen availability.

### Cell-derived EVs

Extracellular vesicles coming from cell-culture supernatants of different immunoregulatory cells would be the choice to modulate further the immune response triggered by alloantigen presentation. One of their main benefits would be to possess a stable phenotype that, contrary to cells, is not subject to further changes or alteration by the milieu. There are three main cell types being studied so far for the production of immunomodulatory EVs: regulatory T (Treg) cells, MSCs, and APCs, mainly DCs.

### Regulatory T cells EVs

As detailed information about Treg EVs and graft rejection can be found in the paper from Lesley Ann Smyth et al. published in this same issue, we will not discuss further this source.

### Mesenchymal stem cells EVs

Mesenchymal stem cells were thought to mediate tissue repair and regeneration through replacement of injured cells by MSCs themselves. Lately, it has been found that rather than proliferating, MSCs promote the secretion of immunomodulatory cytokines and trophic factors in response to damage signaling, encouraging proliferation, and limiting apoptosis of the injured tissue (45, 48, 100). Recently, MSCs were found to secrete EVs, which would mediate MSCs’ signature effects (101–106), although there is some controversy regarding their full efficiency (107).

Mesenchymal stem cells-derived EVs have been used for the treatment of kidney, cardiac, and brain injuries showing regeneration and protective effect against injury, mainly at a paracrine level, and thought to be mediated through cytokines, growth factors and miRNAs delivered by EVs secreted by MSCs (47, 51, 108). In the context of transplantation, some studies have shown promising results, being the most outstanding the study reported by Kordelas et al. in which the infusion of MSC-derived EVs was able to treat a patient refractory to conventional IS therapy in GvHD (109). More information about MSC–EVs can be read on the study by Franquesa et al., in this same issue.

### Dendritic cells EVs

Since Raposo et al. demonstrated in 1996 the presence of MHC molecules in EVs secreted by B cells (110), APC-derived EVs as source of alloantigens has gained enormous interest. Later, studies focused on the description of the cargo molecules present in EVs coming from DCs indicated that DC-derived EVs were not only carrying class I and II MHC molecules, but also accessory molecules involved in T cell co-stimulation (81, 87, 111). As mentioned before, some groups demonstrated EVs were able to engage T cells through direct presentation (96, 112). Others suggested that exosomes required to be captured by DCs to induce an immune response by indirect presentation (94, 95). Moreover, content of MHC molecules in EVs was proven to be sufficient for effective and potent cross-presentation by host APCs. In this sense, it was reported that exosomes from tumor cells could trigger cross-priming of specific antitumor cytotoxic T lymphocytes (113, 114) and DC-derived EVs could induce tumor rejection in mice (68) and in human (115–117). Besides their use as cell-free vaccines in antitumoral therapies, DC-derived EVs have been studied as alternative therapies to induce tolerance in autoimmune diseases and in the transplantation setting.

On one hand, there are strategies focusing on strengthening the immunoregulatory properties of the EV-producing cells. For instance, several approximations engineered DC-derived-exosomes expressing Fas-L or IL-4-transduced BMDC-derived-exosomes, which were used as alternative treatments in models of delayed-type hypersensitivity (DTH) and collagen-induced arthritis (CIA) in mice. These approximations managed to delay the onset and severity of these immune-related diseases (118, 119). In a similar way, exosomes from IL-10-treated BMDCs or transduced with an adenovirus expressing IL-10 suppressed DTH responses (120).

Nevertheless, some effect was reported with mock exosomes, so immature DCs were suggested to secrete exosomes with regulatory properties. In fact, allogeneic exosomes from immature DCs can modulate the rejection of heart allografts (112). Therefore, it is important to consider that the activation state of the DCs producing EVs, may determine the immune response that these EVs will evoke in the host [(68, 112, 121), p. 200; (96)].

In the context of transplantation, donor EVs derived from immature Bone Marrow DC (BMDC) have been used as source of donor MHC antigens in animal models of heart and intestinal transplantation. A single iv administration of donor immature BMDC-derived EVs (imDex) prior to intestinal transplantation in a rat model reduced the host’s anti-donor cellular response, induced the generation of regulatory T cells, and temporally prolonged allograft survival (122). Interestingly, the double infusion of donor imDex before heterotopic heart transplantation prolonged allograft survival in a donor-specific manner (112). This effect was accompanied by a decrease in graft infiltrating leukocytes, a reduction of IFN-γ mRNA expression in the graft, and a decrease in the anti-donor cellular response post-transplantation.

Also, the combination of EV infusion along with non-specific immunosuppressive therapy to favor a tolerogenic microenvironment has also been tested in heterotopic models of heart transplantation (97, 123). Donor imDex administered post-transplantation in combination with suboptimal doses of the immunosuppressive drug LF15-0195 induced donor-specific tolerance, long term allograft survival, and delayed chronic rejection (97). Furthermore, the combination of rapamycin and donor imDex injected before and after transplantation promoted donor-specific tolerance, induced the generation of regulatory T cells and prolonged allograft survival, this time in a mouse model (123). In both cases, donor-specific tolerance was demonstrated to be transferable to naïve recipients, thus allograft survival would be mediated by cellular tolerance.

## Critical Points

The use of EVs for tolerance induction share some of the critical points identified before in experimental cell therapies. As in these approaches, the route of administration, timing and frequency of administration, and the dose are some of the unsolved problems in EVs therapy.

Extracellular vesicles may be administered through several routes for tolerance induction, depending on the specific pathologies. For instance, intra-articular injection of anti-inflammatory exosomes has been used in rheumatoid arthritis patients [reviewed in Ref. (124)]. Also, intranasal administration has been tested in mice models of allergy (125). In the case of experimental transplantation, and similarly to cell therapeutic approaches, intravenous administration is the route of choice for injecting EVs in most of the experimental procedures.

The fate of (intravenously) injected EVs is still under discussion. It has been described that the expression of integrins, adhesion molecules, lipids, and other molecules on EVs contribute to the attachment and fusion of the injected vesicles to “acceptor” cells (126–130). *In vitro* experiments have shown that internalization of EVs is an active process (inhibited by cytochalasin D, EDTA, or low temperatures among others). *In vivo* imaging experiments revealed that intravenously administered EVs rapidly disappear from circulation and are firstly found in liver and lungs (131). Partially confirming these results, *in vitro* capture of EVs has been observed by liver and macrophage cell lines (98), and also described in splenic and peripheral blood DCs (127, 132). It is thus tempting to speculate that liver and spleen resident cells will be the main targets of intravenously injected EVs, and thus these cells will initially conduct the ongoing response (95).

A special mention needs to be paid to the different types of vesicles and their cellular origin. Obviously, different types of vesicles (such as apoptotic bodies and exosomes) from different cellular origins (MSCs, DCs, and others), or even the activation state of the EV-producing cells (for instance, immature versus mature DCs) will produce a specific response on target cells (and tissues) most probably through different mechanisms. In the past years, an outstanding effort has been made to elucidate the mechanisms of action of EVs. Recently, Robbins and Morelli reviewed the regulatory effect of EVs (from different cell origin) in the immune system (133). Yet, their regulatory effects *in vivo* are largely unknown, especially in humans. Probably, one of the first demonstrations of the *in vivo* regulation by EVs is the reported effect of MSC-derived EVs to treat a patient refractory to conventional IS therapy in graft-versus-host disease (109). Apparently, the mechanism of action was an impaired capability of the patient’s PBMCs to release pro-inflammatory cytokines in response to the EV treatment. Speculatively, this effect could be attributed to a higher IL10/IFNγ ratio in the infused EVs, although other mechanisms (generation of Tregs, miRNA regulation) may also contribute to the observed anti-inflammatory effect. The definition of a given type of vesicles to specifically apply for therapeutic purposes will undoubtedly depend on the pathology, mechanism(s) of action, and the feasibility to obtain sufficient amounts of EVs under GMP conditions to conduct the therapeutic approach.

Certainly, the definition of therapeutic doses is another important issue to be solved for the use of EVs in therapeutic applications. Interestingly, in SOT studies in mice and rats, a common observation seems to point to 10–25 μg of EV-associated protein per dose as the optimal quantity leading to increased survival of the graft (Table 1) (97, 121–123). Remarkably, exceeding or decreasing this quantity could only reproduce the results in part. It is noticeable though that this specific dose seems to work even when different concomitant IS regimes were used and also in spite of injecting different number of doses and at different days pre or post-transplantation. Given that the protocols used to enrich EVs do not preclude the presence of contaminant proteins in EV preparations, protein determination does not seem to be an accurate method to define the actual dose of EVs used. Beyond differences among several laboratories, this could also lead to variability among different preparations or batches in a given lab. Debates on the adoption of quantitative standards applicable to different laboratories, together with nomenclature of EVs, are underway in international forums.

**Table 1:** **EV treatments in transplantation-related settings**.

Author	Model	EV origin	Qty^c^	EV infusion (d = days)	IS^d^	Conclusion
Pêche et al. (112)	Rat heterotopic heart TX^a^	imDex^b^	10 μg	d14 pre-TX d7 pre-TX	None	Short term survival, donor-specific
Pêche et al. (97)	Rat heterotopic heart TX	imDex	25 μg	d1 post-TX d6 post-TX	LF15-0195	Long term survival, donor-specific, transferable
Yang et al. (122)	Rat intestinal TX	imDex	20 μg	d7 pre-TX	None	Short term survival
Li et al. (123)	Mouse heterotopic heart TX	imDex	10 μg	d7 pre-TX, d0, d7 post-TX	Rapamycin	Short term survival, donor-specific, transferable
Kordelas et al. (109)	Human refractory GvHD	MSC-EVs	1 Unit	Several doses	Steroids	Reduced clinical GvHD

*^a^TX, transplant*.

*^b^imDex, immature dendritic cell-derived EVs*.

*^c^Qty, quantity of EVs administered*.

*^d^IS, immunossuppressive drug regime*.

Scaling up this hypothetical working dose of 10 μg to a 60-kg human being would result in approximately 30 mg of EV-associated protein per dose. Data regarding this specific point is yet scarce. In the first phase I clinical trial using DC-derived EVs for melanoma vaccination, intradermal or subcutaneous EVs were injected at doses set up based on the concentration of MHC molecules in the EV preparations (116). More related to the induction of tolerance, Kordelas et al. defined one EV unit as the quantity of EVs recovered from 4 × 10^7^ MSCs after 48 h in culture. The NTA analyses of this supernatant revealed a range of 1.5–3.5 × 10^10^ vesicles per unit and between 0.5 and 1.6 mg of protein (109). Further work is needed to define the optimal dose, number, and frequency of administrations for a given therapeutic situation. These may well benefit from the development of new methods aiming at an accurate quantification of EV preparations (134–136).

Thus, a number of additional questions need to be fully answered regarding EV therapy. Apart from the source of EVs, the optimal route, dose, and frequency of administration, other issues such as the standardization of EVs isolation/enrichment or quantitative issues will be necessarily solved in the incoming years. To date, most of the procedures for EVs isolation are based on differential ultracentrifugation. Other methods are based on the capacity of several precipitating agents to favor EV selective enrichment. Most of them though, do not preclude other proteins to be co-enriched with EVs. Further developments on size-exclusion chromatography and immune-based selection of EVs will contribute to reduce the presence of non-vesicular proteins and to improve EV preparations.

Despite these unsolved issues, EV therapy may have some advantages over cell therapy approaches. These include, among others, a non-tumorigenic potential (one of the main concerns on MSC therapy), and the possibility of sterilization by filtration, the capacity of EVs to cross tissue barriers (such as the blood–brain barrier) (137), or the fact that EVs cannot be influenced by the surrounding milieu (138). Some of these advantages may possibly favor the definition of less restrictive regulatory conditions, allowing an easier implementation of EV therapies.

## Concluding Remarks

After a twentieth century, in which the development of IS has permitted outstanding advances in the field of transplantation, the next challenge of this discipline is a graft for life (139), that is, preventing chronic rejection of engrafted organs. Different approaches are exploring how to achieve this goal, including combination of organ and bone marrow transplantation (140). Hypothetically, discontinuation of IS after an initial acute phase would contribute to reduce side effects, thus importantly improving life expectancy after transplantation.

Given their particular characteristics, the contribution of EV therapy in organ transplantation for tolerance induction may be advantageous compared to other approaches in development, such as cell therapy. Together with their potential as drug-delivery carriers, cancer therapy, or in biomarker discovery, using EV strategies in tolerance induction will undoubtedly be one of the future areas of interest in biomedicine and biotechnology.

## Conflict of Interest Statement

The authors declare that the research was conducted in the absence of any commercial or financial relationships that could be construed as a potential conflict of interest.
